# Roadmap of computer-guided bi-axial alveolar distraction osteogenesis in the posterior mandible: neurosensory safety of double scan virtual protocol

**DOI:** 10.1186/s12903-026-08516-y

**Published:** 2026-05-13

**Authors:** Amany M. Alryess, Lydia N. Melek, Yehia El-Mahallawy

**Affiliations:** Oral and Maxillofacial Surgery Department, Faculty of Dentistry, Alexandria University, Alexandria, Egypt

**Keywords:** Alveolar ridge augmentation, Mandible, Distraction osteogenesis, Surgery, Software, Computer-assisted, Computer-aided design, Computer‐aided manufacturing

## Abstract

**Objective:**

The study aims to assess the applicability and safety of using a fully digital workflow for the application of bi-axial alveolar distraction osteogenesis in the posterior mandibular region.

**Materials and methods:**

This study included 7 patients with vertically deficient posterior mandible managed with the utilization of a bi-axial distractor and the implementation of a double scan computer-guided virtual planning protocol. The neurosensory safety of the procedure was assessed using an inferior alveolar nerve conduction test. Radiographic assessment was conducted for vertical bone gain, degree of alveolar segment tilt, bone density, and crown to available bone for implantation ratio appraisal.

**Results:**

Full neurosensory recovery was documented for all patients one month after the activation period. Vertical bone gain reported a statistically significant difference across the radiographic follow-up (*P* = 0.001). The mean bone density showed a statistically significant difference throughout the study period (*P* = 0.001) while the degree of tilt of the distracted segment showed insignificant differences (*P* = 0.072). Crown-to-available bone for implantation ratio showed a statistically significant change post-distraction (*P* = 0.018).

**Conclusion:**

Virtual planning and computer-guided workflow for the application of vector-dictating distraction osteogenesis in the posterior mandible showed a predictable application, ease of operation execution, neurosensory safety, and maintenance of the transport segment buccolingual orientation.

**Trial registration:**

Trial was registered at clinicaltrials.gov [NCT05602909/ 2022-10-14].

**Supplementary Information:**

The online version contains supplementary material available at 10.1186/s12903-026-08516-y.

## Introduction

Vertically deficient posterior mandibular alveolar ridges pose a challenge for the operator during implant placement, which requires a preparatory stage to provide sufficient surrounding bone to house the dental implant [1]. A plethora of surgical procedures were advocated, such as onlay block grafting, sandwich osteotomy, and guided bone regeneration. Nevertheless, donor site morbidities are unavoidable in addition to the risk of graft failure [2–4]. Also, nerve repositioning techniques were tried, and it does come with a myriad of drawbacks, such as postoperative numbness that may last for a few months, risk of mandibular fracture, and incorrect ratio between the crown to the implant due to the lack of vertical augmentation [5, 6].

On the other hand, vertical Alveolar Distraction Osteogenesis (ADO) increases bone height while simultaneously lengthening the associated soft tissues via histogenesis. The alveolar soft tissue benefits from improved quantity and quality via the histogenesis process, which avoids the necessity for additional peri-implant soft tissue procedures, which are inevitable with other bone augmentation techniques. The ADO technique benefits from the high vascularity of the created interpositional gap located between two viable bones, which enhances bone formation due to the rich medullary blood supply [6, 7].

Despite its favorable osseous and soft tissue performance, ADO comes with several drawbacks. Accurate control of the distracted segment vector is an arduous procedure, especially in the posterior mandible [8]. The innate resorption pattern in this quadrant causes poor device trajectory, as the device is positioned on the buccal exterior of the alveolar bone. Furthermore, local muscle pull, inappropriate device position, and inaccurate osteotomy cuts further affect the distraction direction [8].

Researchers have explored several tools to assist in guiding the transport disc to its intended final position, facilitating more precise and multidirectional bone growth [8, 9]. Robiony et al. presented an extraosseous bi-axial distraction system in the mandible, which permits the control of the desired distraction vector and corrects the transported segment’s horizontal position [8]. Dai-Ying et al. utilized the bi-axial distractor in the anterior alveolar region and revealed that this bi-axial contraption could vertically distract the segment and position it in a preferable labial orientation, all while achieving a histologically mature and densely mineralized bone [9].

With the advent of Computer-Aided Designing/ Computer-Aided Manufacturing (CAD/CAM) know-how, static patient-specific bone-supported surgical guides were recently used to guide osteotomy placement, limiting the dangers indicated above, yielding positive results, and opening the door for additional research [10, 11]. Even though it is feasible to use freehand techniques for most grafting procedures, software planning enlightens the surgeon about the proposed cut in relation to the bone and the Inferior Alveolar Nerve (IAN) spatial position. In the endeavors taken to achieve a surgical procedure with minimal morbidity and complications, Resnick outlines that the implantation of virtual preoperative planning could not only allow for a reduction of the intraoperative time and uncertainties during the surgical procedure but also allow a safe positioning of the osteotomy in relation to the nerve [12].

Hence, this study aimed to introduce a fully virtual workflow for the planning, designing, and subsequent implementation of a bi-axial extraosseous alveolar distraction device in the posterior mandibular region with emphasis on the neurosensory safety of the procedure. The presumed null-hypothesis was that the computer-guided double-scan protocol does not improve neurosensory outcomes or bone orientation compared with conventional ADO. The secondary outcome of this study was to weigh the effectiveness of the bi-axial device in the avoidance of the distracted alveolar segment lingual tilt, and to evaluate the gain in the postoperative bone density.

## Patients and methods

### Study design

The study was conducted as a prospective single-arm phase-I clinical trial, based on the CARE guidelines (http://www.care-statement.org/). This study was approved by the local research ethics committee (IORG0008839) and was registered in clinicaltrials.gov [NCT05602909/ 2022-11-1]. The doctrines of the Helsinki Declaration for medical research involving human subjects were followed during the implementation of this study, and all of the involved participants signed a written informed consent before embarking on the study. Based on a sample size estimation, seven patients who presented with atrophic posterior mandible requiring ridge lengthening were involved in this study. The sample size was estimated based on assuming a 5% alpha error and 80% study power using the difference between the two dependent means (SD) of bone density for patients treated by osteogenic alveolar distraction, using the highest SD = 179.47 to ensure enough study power. (Gpower 3.1.9.7) [13–15].

Recruitment was performed from those admitted to the outpatient clinic of the Oral and Maxillofacial Surgery Department at the Faculty of Dentistry, Alexandria University. Adult patients, ranging from 35 to 50 years, seeking fixed prosthetic rehabilitation in the posterior mandible were included in this study. The remaining posterior atrophic mandible must have a minimum alveolar height distance of 6-mm from the alveolar crest to the upper border of the canal, and a minimal alveolar width distance of 5-mm. The recruited patients agreed to be committed to all follow-up visits for a minimum period of 4 months postoperatively and with adequate oral hygiene maintenance. Patients receiving radiotherapy, chemotherapy, or bisphosphonates, and those with bone diseases, any habits that might retard healing, such as heavy smoking or alcoholism, or a history of any grafting procedure at the designated area were excluded [16].

A detailed medical and dental history was taken of all the patients, followed by a clinical examination to ascertain the adequateness of the inter-arch space, gingival biotype, oral hygiene, and conditions of adjacent and opposing dentition. Participants were radiographically assessed using a Cone-Beam Computed Tomographic scan (CBCT) *(R0)* (Ingenuity Core; Philips Medical Systems, Cleveland, OH).

**Fig. 1:**
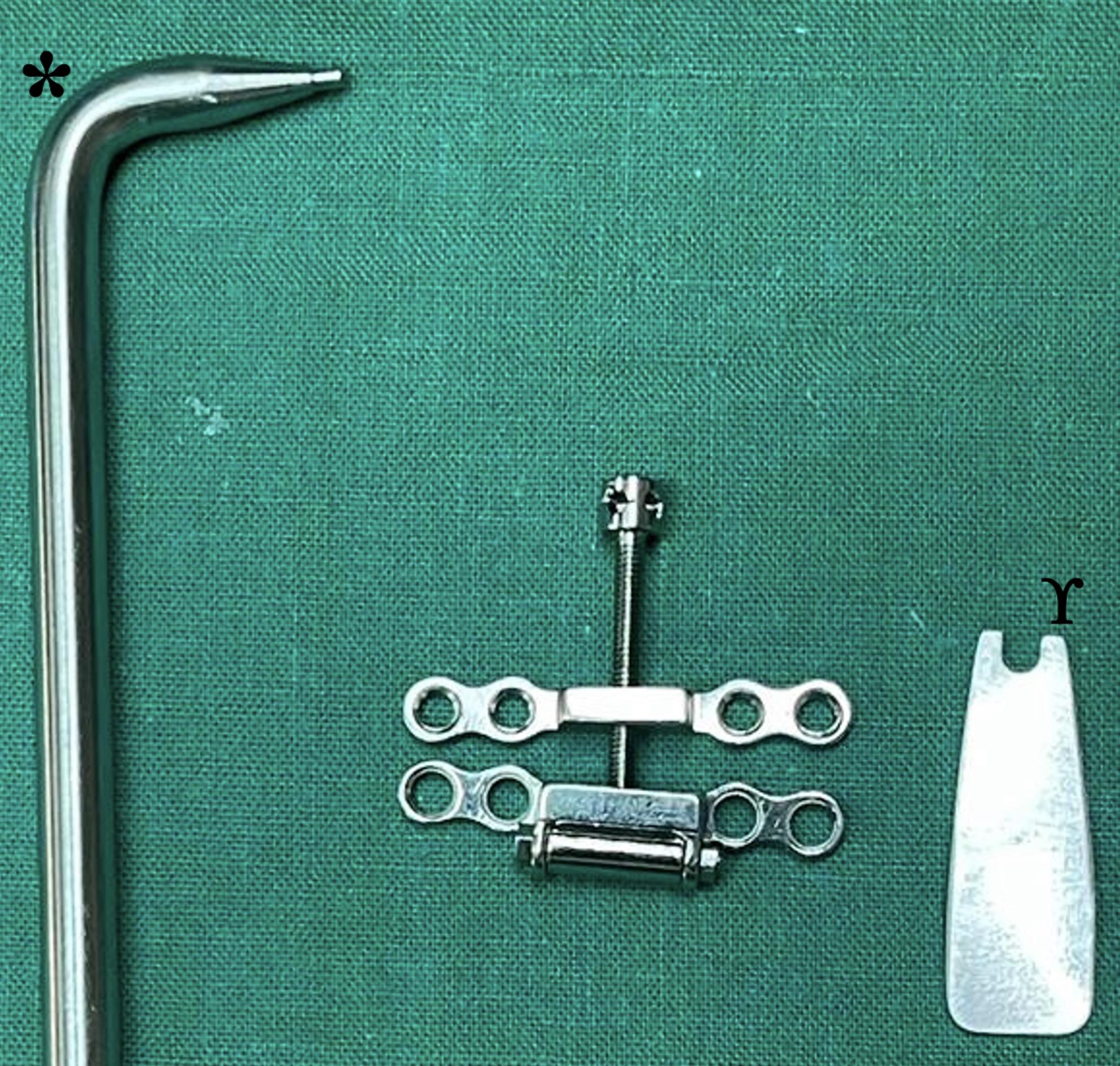
Bi-Axial extraosseous alveolar distraction device. The system comes with an activation key (*) and a hinge-lock key (ϒ)

The Bi-axial extraosseous distractor is a titanium device with upper mobile and lower fixed miniplates and a central rod for vertical distraction. One turn of the key causes plate separation by 0.5 mm. The device encompasses a distinctive hinge at the fixed plate, which causes the mobile plate to rotate in a buccolingual direction but within the outer circumference of the fixed lower plate (Arabic Engineers for Designs and Medical Instrument, Cairo, Egypt) (Fig. 1).

### Double scan virtual planning protocol and guide fabrication

Virtual planning was accomplished using the preoperative CBCT and segmentation software with a surface-based rendering for surgical planning and simulation (Materialise, Leuven, Belgium). The invented virtual planning protocol is constructed on a double scan protocol for meticulous execution and accurate operative implementation. The first part of the double scanning virtual planning protocol is a radiographic scan, which is initiated by uploading the two-dimensional CBCT data in Digital Imaging & Communications in Medicine format (DICOM) to a segmentation software (MIMICS; Materialise) for automatic thresholding and 3D-model creation of the mandibular bone and dental tissues. The outline of the Inferior Alveolar Canal (IAC) was manually segmented to create a Three-Dimensional (3D) IAC part. The generated 3D-models of the mandible and the IAC were uploaded to a designing software (3Matic; Materialise), on which a provisional bone-cutting guide and a guide for the outline of the canal were created using a 2-mm offset. The bone-cutting guide was designed 2 mm away from the 3D model of the IAC. Both guides and the mandible model were transferred in a Standard Tessellation Language (STL) format to a specialized software (NETFAB, Autodesk, CA, USA) for 3D printing of the selected parts using Fused Deposition Modelling technology (FDM) (Creality 3D Printer CR-10 S4; Creality 3D, Shenzhen, China). The IAC outlining guide was seated on the printed mandible, and a pencil was used to draw the projection of the IAC on the buccal surface of the bone model. Additionally, the cutting guide was seated on the printed mandible, and the provisional outline of the bone cuts on the printed mandible was outlined. The bi-axial distractor was then seated on the printed mandible, and using the outlines drawn on it, the basal holes of the distractor were confirmed to be below the projection of the IAC, and the movable plate holes were confirmed to be within the boundaries of the cutting guide and more than 2 mm above the IAC projection. This was followed by the drilling of the distractor holes in the printed mandible.

**Fig. 2:**
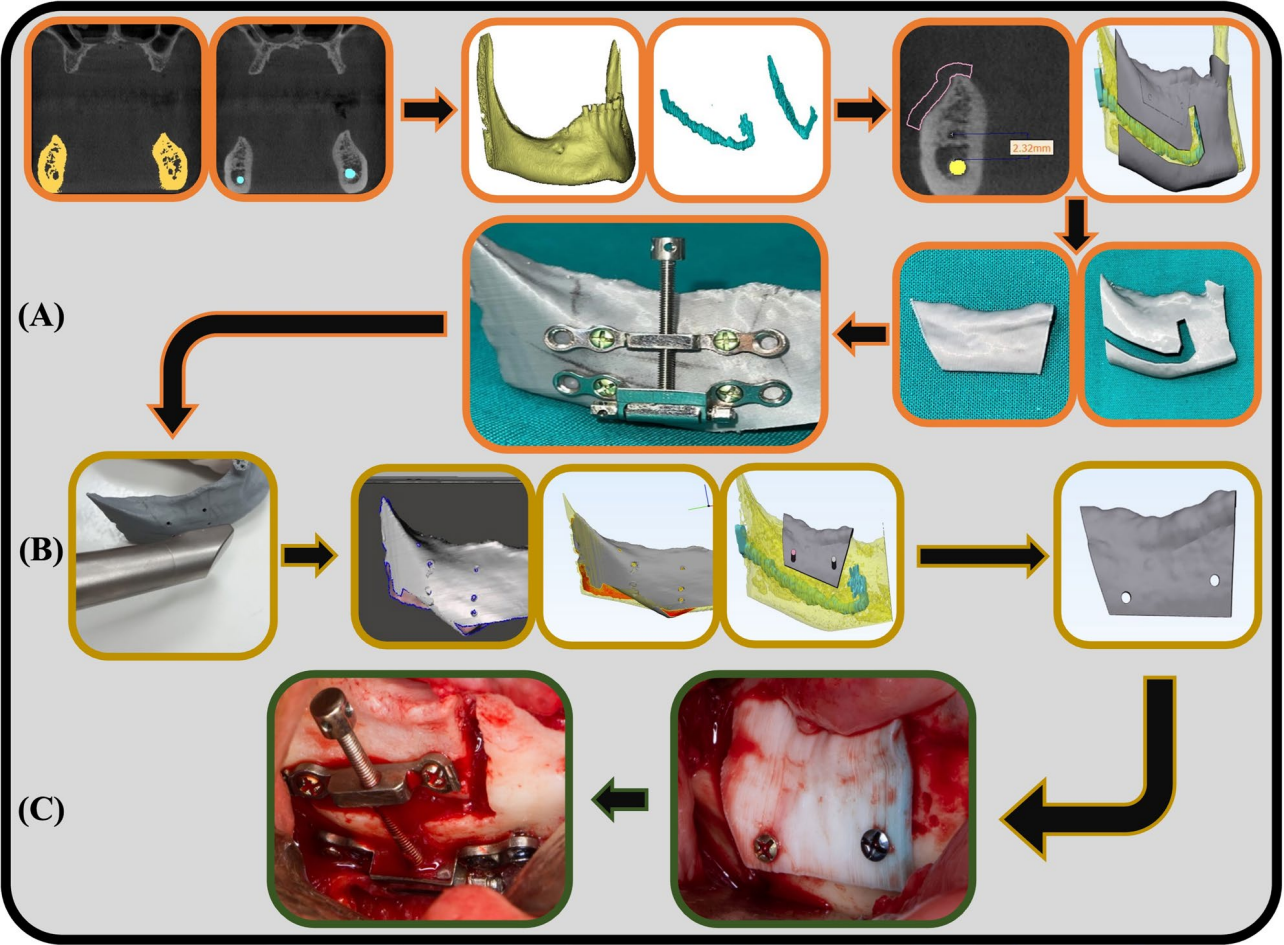
Double Scan Virtual planning protocol for application of Bi-Axial extraosseous alveolar distraction device in vertically deficient posterior mandibular region. The first radiographic scanning part of the virtual plan is presented in segment (**A**), which consists of mandible and IAN thresholding and 3d-model creation, IAN outlining and provisional osteotomy guides designing and 3D-printing, nerve and osteotomy outlining, and provisional distractor fixation. The second optical scanning part is presented in segment (**B**), which consists of model scanning, superimposition, final guide designing, and osteotomy cutting guide printing. Segment (**C**) shows osteotomy cutting guide intraoperative fixation and device installation

The second part of the double scanning virtual planning protocol is an optical scan for the 3D-printed mandible with the provisional drilled distractor fixation boreholes, which was conducted using Medit i700 (Medit. i700 Intraoral Scanner: https://www.medit.com/i700). The scanned mandible with the distractor holes was superimposed on the original mandible on the designing software using a point-to-point registration tool (3Matic; Materialise). The outline of the cutting guide was modified according to the position of the movable plate holes. This would ensure that the placement of the final screws is located within an ample amount of transport segment bone circumferentially, all while maintaining neurosensory safety for the IAN. The position of the movable plate drilling holes was subtracted from the cutting guide. Finally, the final *Osteotomy Cutting Guide* with the distractor’s movable plate boreholes was FDM-3D printed (Fig. 2).

### Surgical procedure

All patients were operated under general anesthesia using nasal Intubations. The surgical procedure commenced by elevation of a mucoperiosteal flap of a buccally placed paracrestal incision line posterior to the mental foramen. Subsequently, the *osteotomy cutting guide* was seated and fixed to the mandible, and three osteotomies were performed using a piezo surgery device (ACTEON, Merignac, France) to shape the bone into a trapezoidal crestal segment. The surgical guide was also used to drill the distractor screw sites in the movable plate, to install and fix the ADO device to its preplanned position using mono-cortical mini screws on either side of the distraction rod. The basal plate was fixed using a trans-buccal approach to minimize manipulation. The lower hinge was used to adjust the buccolingual orientation of the movable segment and keep it within the outer circumference of the lower plate. The hinge was tightened in the desired place to snug it during the activation and consolidation period. Finally, the device was activated to test for the widening of the bony gap, and the wound was closed primarily.

### Distraction process description

A 5-day postoperative *Latency Period (LP)* was maintained in all cases before the activation period and bone elongation initiation at a rate of 0.5 mm/day to achieve painless stretching and appropriate adaptation of alveolar soft and hard tissues until the required height had been achieved. A slight overcorrection of 2–3 mm was planned in each case (*AP*). After that, the distractor was left in place for an additional bony *Consolidation Period (CP)* of 3 months.

### IAN neurosensory recovery assessment

For the IAN neurosensory recovery assessment, the technique described by Jaaskelainen et al. [17] was utilized, and patients were tested pre-operatively *(T0)*, after activation *(T1)*, and 1-month post-activation *(T2)*. The IAN was recorded orthodromically using surface electrodes. The active recording surface electrode was placed in front of the temporomandibular joint about 3 cm anterior to the tragus, the reference electrode was placed on the skin overlying the zygomatic arch, and the ground electrode was wrapped around the upper arm. A bipolar surface-stimulating electrode with the cathode placed on the mental nerve at its exit from the Mental Foramen (MF) was used. The position of the foramen was estimated by the location of the premolar and by palpation. Electrical square-wave pulse stimuli with a duration of 0.2 milliseconds (ms) and an intensity of 3–5 times the sensory threshold were applied, ranging from 6 to 15 mA. Sensory IAN *Onset Latency (OL)*, *Amplitude*, and *conduction velocity (CV)* were measured in (ms), microvolts (µV), and meters per second (m/s), respectively.

### Radiographic postoperative assessment

**Fig. 3:**
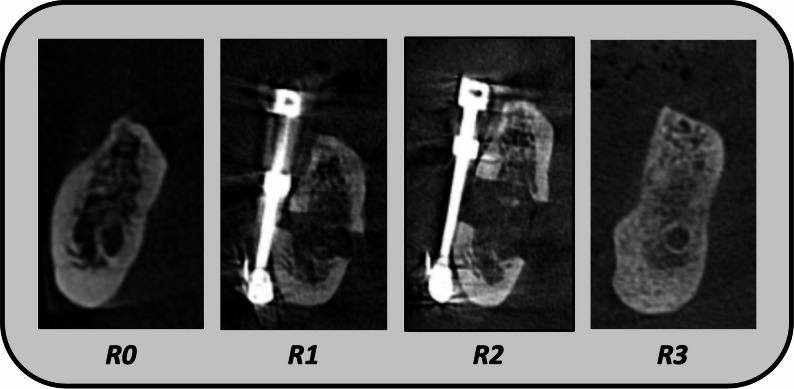
A complete description of the radiographic timeline of the study. R0, Preoperative CBCT. R1, pre-activation CBCT. R2, Post-Activation CBCT. R3, Post-Consolidation CBCT

**Fig. 4:**
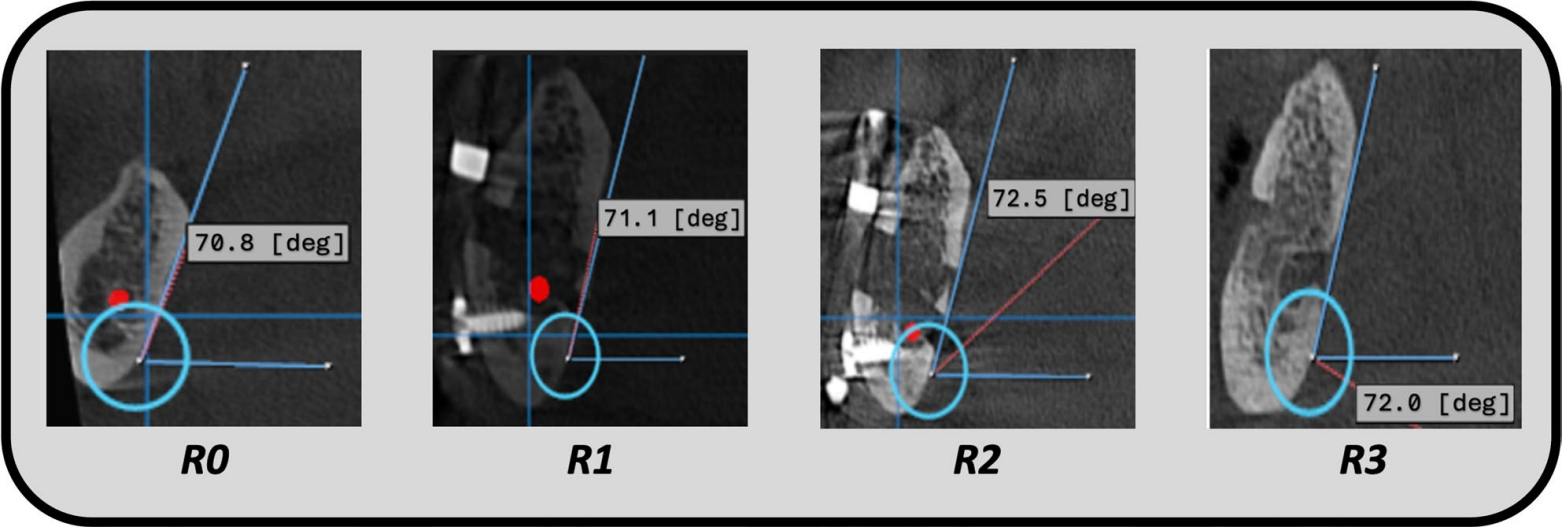
Lingual displacement tilt angle assessment across the radiographic timeline of the study. The tilt angle is calculated between the basal bone and movable segment from the lingual side in the cross-section view of the CBCT record

For a comprehensive radiographic assessment, pre-activation *(R1)*, post-activation *(R2)*, and post-consolidation *(R3)* CBCT records were obtained. All of the obtained scans, along with the preoperative one, were obtained through a calibrated, standardized machine. The post-consolidation scan was obtained 3 months after the end of the activation period. A complete description of the radiographic timeline is presented in Fig. 3. The *Vertical Bone Gain* was measured from the crest of the ridge to the upper border of the IAC. This was measured by assigning 6 different cutting lines on the panoramic view in each CBCT scan, and in the corresponding cross-section view, the length was measured. The mean value was obtained for each case. The *Alveolar Segment Tilt Angle* was calculated between the basal bone and movable segment from the lingual side in the cross-section view of the CBCT record (Fig. 4). The *Crown to Available Bone for Implantation ratio* (C: ABI) was assessed in the post-consolidation period *(R3)* and compared to the preoperative value *(R0).* C: ABI assessment was done by measuring the inter-arch space clinically in relation to the available bone for implant radiographically [18]. The Mean Bone Density value was obtained by assigning 6 different similar cutting lines on the panoramic view in each CBCT scan, and in the corresponding cross-section view, the Region of Interest (ROI) was assigned with a dimension of approximately 3*3, and the value was obtained. The mean ROI of the 6 assigned trajectory lines was calculated.

### Statistical methods

After data tabulation, statistical analysis was performed using IBM SPSS software, and the obtained values were judged significant at the 5% level. For normally distributed quantitative variables, the paired t-test with ANOVA with repeated measures was utilized, while for the abnormally distributed quantitative variables, the Wilcoxon signed-rank test with Dunn’s Post Hoc Test was utilized.

## Results

The study was conducted on 7 patients with a male-to-female ratio of 0.4:1, and a mean age value of 41.43 ± 5.56 years. All of the enrolled participants have adhered to the distraction protocol and the follow-up post-consolidation period. No clinical complications were encountered throughout the activation and the consolidation period. Demographic and distraction data are presented in Table 1.

**Table 1:** Patients demographic data

*n*	Age	Sex	Side	Planned VBG	AP
1	45	F	R	8 mm.	16 days.
2	40	M	R	6 mm.	12 days.
3	35	F	R	8 mm.	16 days.
4	35	F	L	8 mm.	16 days.
5	40	F	L	8 mm.	16 days.
6	45	M	R	7 mm.	14 days.
7	50	F	R	7 mm.	14 days.

*n* Number; *M* Male, *F* Female, *R* Right, *L* Left, *VBG* Vertical Bone Gain, *AP* Activation period

### Neurosensory recovery outcome

**Fig. 5:**
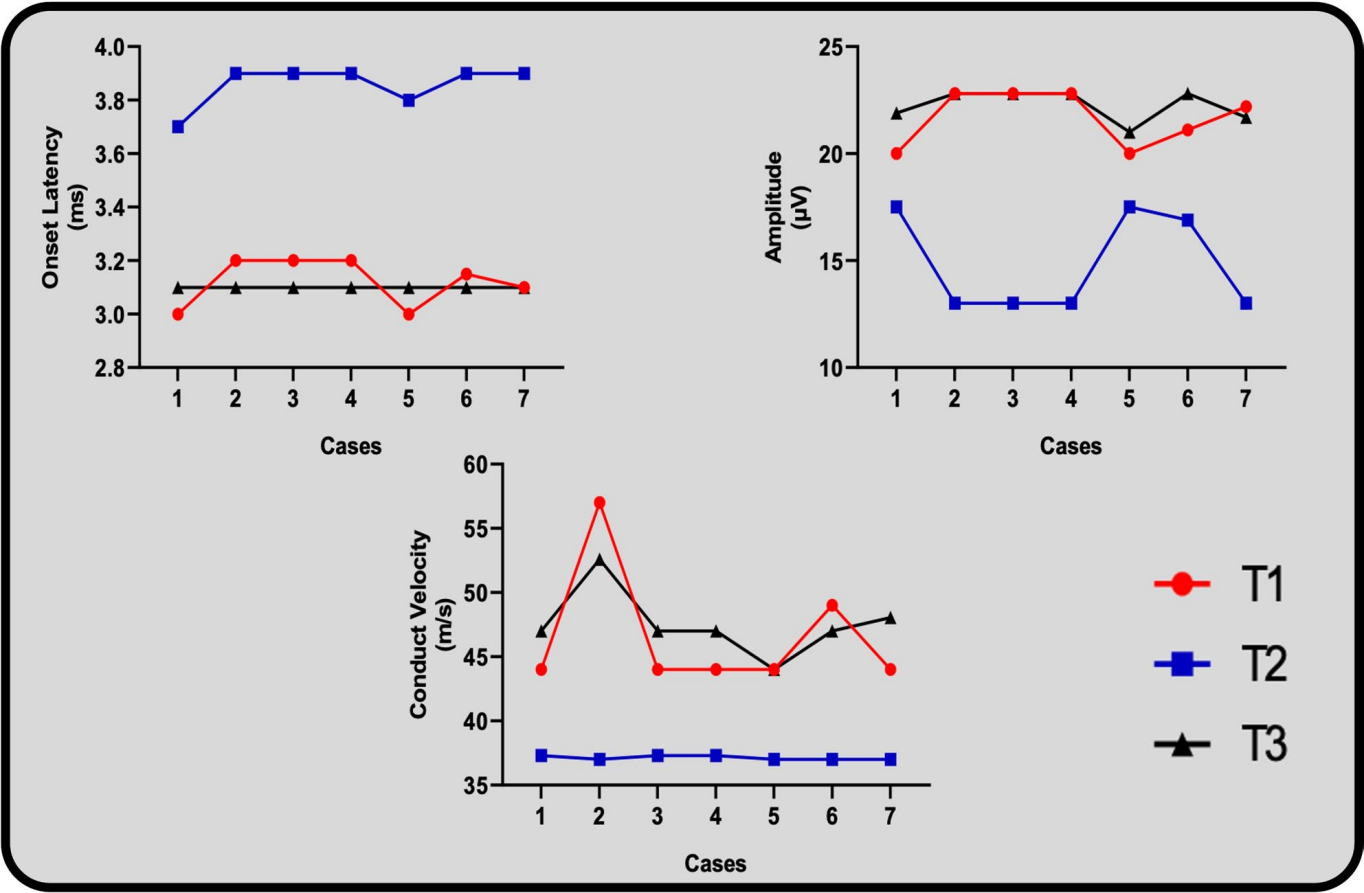
Plotted cases line graph showing the assessed variables in the inferior alveolar nerve neurosensory recovery assessment preoperatively (T0), after activation (T1), and 1-month post-activation (T2). The variables are Onset Latency/ ms, Amplitude/ µV, and conduction velocity/ m/s

In the IAN conduction test, the latency, amplitude, and velocity were recorded, tabulated, and compared to the preoperative baseline result. In all of the above-mentioned parameters, the recorded data reported a statistically significant change through the investigated period. Regarding the latency, a significant increase was reported at T1 compared to the T0. During T2 period, no significant difference was recorded compared to T0 values (*P* = 1.000). Regarding the amplitude, T1 period showed a statistically significant decrease when compared to the T0 values. At T2 period, no significant difference was recorded when compared to the baseline values (*P* = 0.612). IAN conduction velocity showed a statistically significant decrease at T1 compared to the T0 values. At T2 period, no significant difference was recorded compared to T0 (*P* = 1.000) (Table 2) (Fig. 5).

**Table 2:** Inferior alveolar nerve conduction test assessment

(*n*=7)	T0	T1	T2	Test (*P*)
Onset Latency/ mS				
Mean ±SD	3.12 ± 0.11	3.86 ± 0.09	3.10 ± 0.0	F=201.0 (<0.001^*^)
95% CI	3.011-3.22	3.78-3.94	3.10-3.10	
* P0*		0.001^*^	1.000	
* P1*			<0.001^*^	
Amplitude / µV				
Mean ±SD	21.68 ± 1.53	14.80 ± 2.46	22.26 ± 0.80	F=30.866 (0.004^*^)
95% CI	20.27-23.10	12.52-17.08	21.52-22.10	
* P0*		0.023^*^	1.000	
* P1*			0.008^*^	
Conduct Velocity / m/s				
Mean ±SD	46.60 ± 5.81	37.10 ± 0.65	47.52 ± 3.12	F=21.515 (0.001^*^)
95% CI	41.23-51.97	36.50-37.70	44.64-50.41	
* P0*		0.049^*^	0.612	
* P1*			0.003^*^	

*T0* Pre-operatively assessment, *T1* Post-activation assessment, *T2* 1-month’ post-activation assessment, *mS* Milliseconds, *µV* Micro-volt, *m/s* Meter per second, *F* ANOVA with repeated measures, *P* P value for comparing between the studied periods, *P0* P value for comparing between T0 and each other period, *P1* P value for comparing between T2 and T1, *Statistically significant at *p* ≤ 0.05

### Radiographic outcome

The mean preoperative alveolar ridge height was 7.90 ± 0.36 mm, while the mean pre-activation value was 8.40 ± 0.55 mm. The calculated mean ridge height value increased to 15.11 ± 2.42 mm in the post-activation record, showing a statistically significant VBG from the preoperative values (*P =* 0.001). The 3-month post-activation records reported a statistically significant decrease from the R2 values (*P =* 0.037). The changes in the reported alveolar ridge height were statistically significant across the radiographic follow-up period (*p* < 0.001) (Table 3).

**Table 3:** Assessment of vertical bone gain

VBG / mm	R0	R1	R2	R3	Test (*P*)
Mean ±SD	7.90 ± 0.36	8.40 ± 0.55	15.11 ± 2.42	13.54 ± 1.98	Fr= 19.971^*^ (<0.001^*^)
*P0*		0.219	0.001^*^	0.001^*^	
*P1*			0.001^*^	0.001^*^	
*P2*				0.037^*^	

*VBG *Vertical Bone Gain, *R0* Preoperative CBCT, *R1* Pre-activation CBCT, *R2* Post-Activation CBCT, *R3* Post-Consolidation CBCT, *Fr* Friedman test, *P* P value for comparing between the studied periods, *P0* P value for comparing between R0 & ensuing periods, *P1* P value for comparing between R1 & ensuing periods, *P2* P value for comparing between R2 and R3. *Statistically significant at *p* ≤ 0.05

**Fig. 6:**
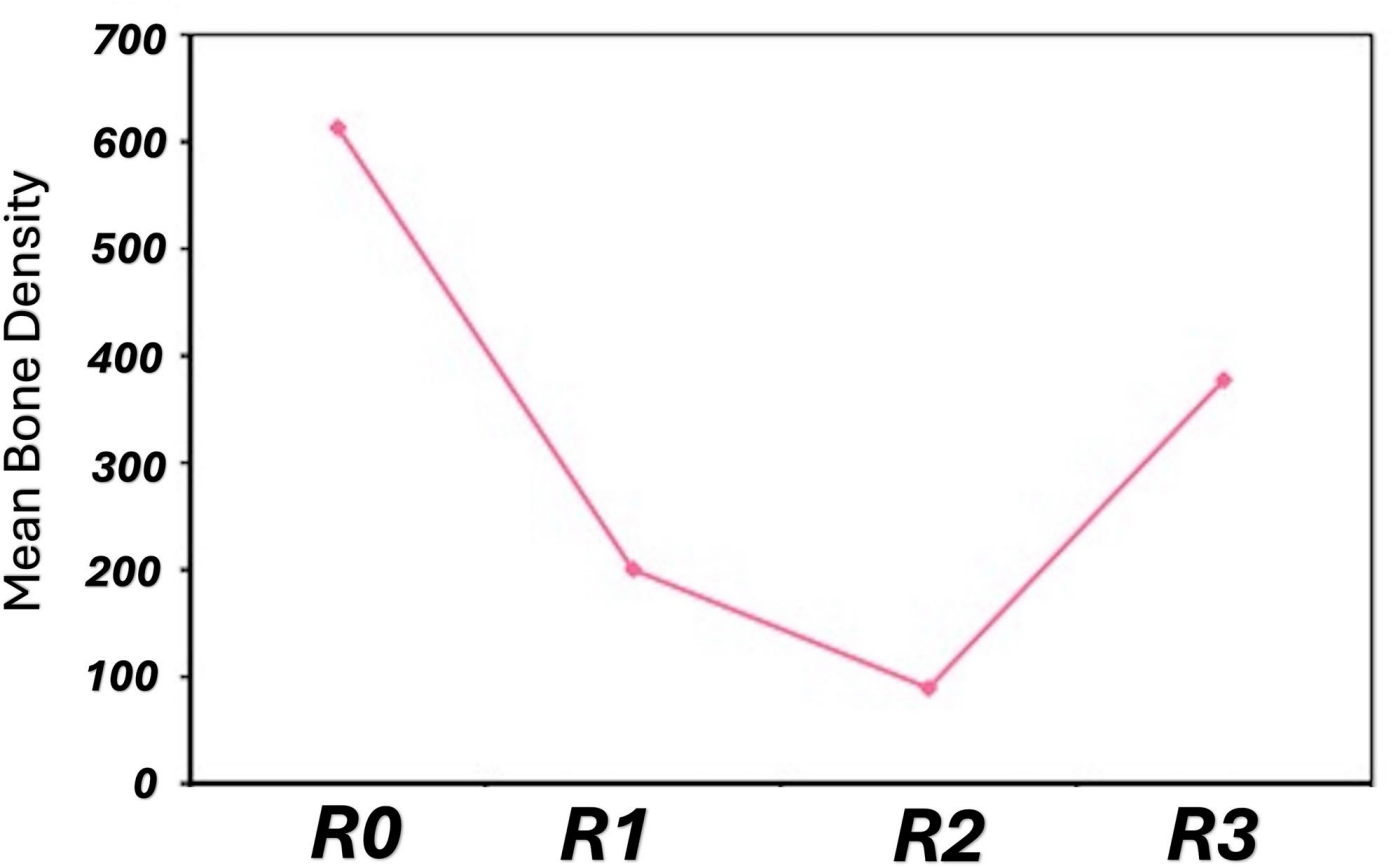
Comparison between the different studied radiographic periods according to the reported mean bone density. R0, Preoperative CBCT. R1, pre-activation CBCT. R2, Post-Activation CBCT. R3, Post-Consolidation CBCT

The mean preoperative tilt degree was 65.41 ± 3.77 °, while the mean pre-activation value was 68.06 ± 2.84 °. The mean post-activation mean tilt degree was 66.0 ± 4.44 °, and the changes in the reported degree of alveolar segment tilt were statistically insignificant across the radiographic follow-up period (*P = 0.072*) (Table 4). Regarding the C: ABI, the pre-distraction value for all of the cases was more than one, with a mean ratio of 2.30 ± 0.25. The mean post-distraction ratio showed a statistically significant change from the preoperative value with a mean of 0.82 ± 0.38 (*P = 0.018*) (Table 5). Regarding the gain in the mean bone density, the immediately postoperative values were 200.3 ± 46.3, which changed to 89.27 ± 37.51 in the post-activation. The reported bone density was significantly superior in the 3 months post-activation scan, with a mean range of 377.34 ± 18.58. The comparison between the preoperative, the immediate postoperative, post-activation, and the 3 months post-activation was statistically significant at (*P* = 0.001) (Fig. 6).

**Table 4:** Assessment of lingual displacement tilt degree

Tilt / °	R0	R1	R2	R3	Test (*P*)
Mean ±SD	65.41 ± 3.77	68.06 ± 2.84	66.0 ± 4.44	65.96 ± 4.23	F=8.015 (0.072)
95% CI	61.92-68.90	65.43-70.69	61.89-70.116	62.05-69.87	
Min - Max	62.0 – 71.0	62.30 – 71.10	62.0 – 72.50	62.20 – 72.0	

*R0* Preoperative CBCT, *R1* Pre-activation CBCT, *R2* Post-Activation CBCT, *R3* Post-Consolidation CBCT, *F* ANOVA with repeated measures, *P* P value for comparing between the studied groups; *Statistically significant at *p* ≤ 0.05

**Table 5:** Assessment of crown: root percentage

C: *R* / %	R0	R3	Test (*P*)
Mean ±SD	2.30 ± 0.25	0.82 ± 0.38	Z= 2.366 (0.018^*^)
95% CI	2.07-2.53	0.47-1.17	
Min - Max	1.97 – 2.59	0.42 – 1.56	

*C:R *Crown-root, *R0* Preoperative CBCT, *R3* Post-Consolidation CBCT, *Z * Wilcoxon signed ranks test, *P* P value for comparing between the studied periods. *Statistically significant at *p* ≤ 0.05

## Discussion

Virtual planning and computer-assisted surgery proficiencies are an integral part of contemporary oral and maxillofacial surgery, ensuring perfect operation execution and minimal morbidity [19]. The study is designed to integrate the utilization of a fully virtual workflow with a double scan protocol for the implementation of a bi-axial extraosseous alveolar distraction device in the atrophic posterior mandibular region. Furthermore, it aims to assess the accuracy of the virtual plan using the IAN neurosensory objective assessment.

The design of the utilized alveolar distractor is based on the track alveolar distractor by KLS Martin. In order to dictate the vector of the alveolar segment in the distraction process, an apical hinge was added to allow differentiation between the apical and coronal plates. Changing the hinge location allows the vector of the alveolar distraction of the coronal segment to be adjusted according to the desirable vector based on the occlusion and the final implant prosthetic position.

To limit other confounding factors, the whole operative procedure was conducted by the same surgeon (AA), where a buccally-paracrestal incision with the preservation of the lingual and crestal mucosal attachments was utilized to preserve an ample blood supply to the transported segment, reducing the resorption and preventing damage to nearby structures. Furthermore, the osteotomy was conducted using soft-tissue-friendly piezoelectric bone cutting tools to achieve a precise cut with preservation of lingual mucosa [20, 21].

The contemporary literature directs attention to the difficulty of distractor device placement, transport segment thinning and fracture, and device fracture as the most recurring complications for the application of ADO in the posterior region of the mandible [18, 22]. The devised virtual planning protocol tackles all of the aforementioned obstacles. The indispensable importance of the double scan implementation was to ensure that the devices’ fixation screw boreholes are located within an ample amount of transport segment bone circumferentially. This would ensure stability during the activation period and retention during the consolidation period. The first provisional cutting guide allowed outlining of the resection margins and visualizing their relation to the screws of the movable plate. Scanning the drilled model allowed the fabrication of the final osteotomy cutting guide with respect to the distractor’s final position. This second optical scan allowed the final osteotomy cutting stent to guide the osteotomy’s spatial positioning with neurosensory perseverance, all while channeling the drilling of the distractor fixation holes in the small alveolar segment with great accuracy. Another advantage of the innovated double scan protocol is the preoperative preparation of the distractor basal plate holes in relation to the projection of the IAC. This would ensure command on various variables that may compromise IAN integrity. Finally, the use of a preoperatively printed mandibular 3d model allowed the preoperative adaptation and preparation of the stock alveolar distractor device, which minimizes the strain on the final fixation screws and lessens the installation time intraoperatively.

According to Rachmiel et al., to avoid breaking the transport segment and ensure proper attachment of the distractor, the height should be at least 6-mm [18]. This is consistent with our study, which reported a mean preoperative vertical length of 7.90 ± 0.36 mm. Furthermore, Ooi et al. suggested that the horizontal cut should leave at least 5 mm of bone above it to maintain enough blood supply to the cut bone segment [23]. Taking this preoperative precaution during patient selection in the current study, along with the utilization of the second optical scan, allowed for favorable conduction and maintenance of the distraction process.

This devised protocol is designated toward the integration of preoperative virtual planning tools and the application of stock distractor devices. The use of a patient-specific distraction device would obviously invalidate the use of the second optical scan; however, machining a multidirectional patient-specific device with two rotatory parts would be troublesome and exorbitant. Although there are many studies done previously on ADO, none utilized virtual planning and computer-aided proficiencies. Robiony et al. assessed the guided placement of the osteotomy line using neurosensory appraisal [12]. To the best of our knowledge, this report is the first to implement the application of a stock multi-axial distractor device through virtual osteotomy placement, double-scanning protocol for computer-guided fixation screws orientation, and correlate it with the neurosensory objective assessment in the atrophic posterior mandibular region.

This study attempted to delineate the accuracy of the proposed virtual planning protocol by testing the IAN neurosensory recovery objectively using the method described by Jaaskelainen et al. [17]. The IAN conduction test reported an increase in latency, a decrease in amplitude, and a decrease in velocity in the post-activation assessment period (T1) when compared to the preoperative valuation (T0). The changes were statistically significant in all 3 variables. Despite that, the 1-month post-activation nerve assessment period (T2) reported a regain in the normal values of latency, amplitude, and velocity compared to the T0 valuation. This could be rationalized as a temporary impairment in the neurosensory performance of the IAN that occurred in the first 2 weeks, which recovered in all cases at the first post-activation month assessment, as shown by the regain of normal neurosensory objective signs.

The fluctuation seen during the early follow-up period may be related to manipulation during the operation and postoperative edema, which caused a little degree of neuropraxia. The favorable rapid regain in normal sensation, as objectively seen, may be attributed to a plethora of measures taken during the conduction of this study. Primarily, the use of virtual planning integrated with a double scan protocol allowed for maximizing the size of the distracted alveolar segment while precisely placing the basal osteotomy above the boundary of the IAN. This procedure started with manual nerve mapping and segmentation, proper delineation of IAN projection, and ended with the utilization of a piezoelectric bone cutting kit to ensure optimal neurosensory security. Another measure taken to decrease the amount of tissue manipulation, especially during the fixation of the lower border plate, is the use of a trans-buccal approach, which resulted in a rapid objective recovery of sensory function. Resnick et al. showed that using computer-assisted surgery improves accuracy, decreases the need for lots of tissue dissection, and limits surgical morbidity [19]. This study correlated the excellent performance of the double scan planning to the favorable and prompt neurosensory recovery outcome.

Regarding the vertical bone gain, the use of ADO in this study reported a statistically significant increase in post-activation alveolar bone length when compared to the preoperative value (*P =* 0.001). A similar outcome was reported by Bianchi et al., who compared ADO with inlay bone grafts. They also reported that the gain in vertical bone height was superior in the ADO group [24]. There is a literature consensus that 10–20% of the vertically gained bone may relapse owing to various factors and depending on the type of technique used [18, 25, 26]. This study opted for a pre-planned over-activation of the distraction device by 2–3 mm to compensate for the expected relapse in the consolidation period. The post-consolidation radiographic record showed a statistically significant decrease in alveolar ridge length when compared to the post-activation scan (*P =* 0.037). Despite that, the decrease in VBG reported a 10.4% relapse with a mean difference of 1.57 mm, which is better than the anticipated preoperative values. Keestra et al. reported that meticulously conducted ADO is more stable and has less degree of alveolar resorption than onlay bone grafts or guided bone regeneration [27]. Altaweel et al. reported a higher relapse rate, with mean post-consolidation and post-activation values of 11.5 ± 1.4 mm and 13.6 ± 1.4, respectively [28]. The reported outcome in this study could be correlated to the meticulous nature of distraction operation execution and the utilization of virtual planning in this study, which lessened operation time and complication rates.

An evident increase in bone density was reported through the distraction periods, which is in agreement with the work of Altaweel et al. [28]. The study documented proper bone consolidation in the distraction gap, which is evident with the substantial increase in bone density. When comparing the preoperatively bone density with the 3 months after alveolar distraction scan, mean values of 613.19 ± 58.81 and 377.34 ± 18.58 were reported, respectively. Türker et al. come with a similar observation, where the formed bone after 3 months was more transparent than the existing bone after a year-long follow-up scan [29]. Computed tomography grey values were used as a relative indicator for bone density appraisal, as they lack the absolute quantitative accuracy of Hounsfield Units in the conventional scans. While grey value analysis owes its own variability, the utilization of a standardized acquisition protocol and calibration for all of the scans could ensure data consistency.

Control of the vector of the transport segment in ADO is challenging, owing to the small dimension of the distracted bone, which is easily affected by various factors. The study utilized a bi-axial distractor contraption in a vertically deficient posterior mandibular alveolar ridge. The measured mean segment tilt degree showed an insignificant fluctuation across the radiographic follow-up, with changes of approximately 3 degrees (*P = 0.072*). The favorable outcome in this study indicates the capability of the multi-directional distractor to dictate the vector of the transport segment and maintain it during the distraction process, negating the effect of other local factors in causing lingual segment displacement.

The innate anatomy of the body of the mandible and the curvature of the submandibular fossa may hinder dental implant rehabilitation in this region. The effect of the mylohyoid muscle on the distracted alveolar segment may be dreadful for the final consolidated bone and the final bone orientation, which may require off-axis implant placement and subpar occlusal load transfer [30]. The implementation of a bi-axial extraosseous distraction device allowed the transported segment’s horizontal position to be guided by the operator and to mitigate other uncontrollable variables. The utilization of a standardized 3-month post-consolidation period, and the procurement of 3D radiographic assessment allowed the study to visually appraise the ability of the device to achieve sufficient retention to ensure that newly formed bone is strong and stable before placing dental implants. The study is innovative in the assessment of the segment displacement degree methodology, as it utilized the lingual plate and the lower border of the mandible trajectories for angle assessment.

Implant placement in the atrophic posterior mandible presents a significant challenge owing to the imbalance in the crown-to-available bone for implantation ratio. ADO enables the use of longer implants with shorter crowns and enhances implant prognosis [18, 31]. A less than, or equal to one C: ABI ratio is required for the implant to be placed with a reliable prognosis and FP-1 prosthetic rehabilitation [18, 32]. In this study, the mean pre-distraction ratio was 2.30 ± 0.25, which significantly decreased to < 1 after distraction (0.82 ± 0.38, *P* = 0.018^*^). The favorable outcome of this study is consistent with the recommendation of Bruhnke et al., who emphasized the ultimate objective of achieving a crown-to-implant length ratio of less than one [32]. Yun et al. conducted a systematic review comparing ADO versus onlay bone grafting. They outline that processes of histogenesis, along with the favorable crown-to-implant ratio, are distinctive features in ADO when compared to other atrophic ridge augmentation modalities [33].

The promising outcomes observed in this report likely reflect a synergetic effect between the digital workflow and rigorous clinical standards. While the ADO protocol provides the technical framework for precision, it is important to consider the role of meticulous case selection and operator expertise in achieving these results. These variables are inherently linked in advanced surgical procedures; however, the absence of a direct comparator group makes it difficult to isolate the precise weight of each variable.

Despite that, ADO in the management of vertically defective alveolar ridges has a few drawbacks, such as the need for rigorous patient compliance, which may cause loss in the follow-up period. The study did consider this during sample size estimation, and proper patient selection prevented this from occurring in the current study. Furthermore, all of the operations were conducted under general anesthesia. This was a preference taken in this study to ensure impeccable execution.

Although a bone-supported computer-generated guide affects the intraoperative visibility and prohibits any alterations to the original plan if mandated, this was overhauled by the double scan protocol. Furthermore, the application of computer-guided workflow owes its own decision-making bias. The transfer of a virtual preoperative plan intraoperatively limits operative variables and makes the conduction of a randomized trial conduction incomprehensible.

The favorable outcome of this feasibility study petitions for a controlled trial with a larger cohort in order to negate measurement and operator bias. Furthermore, the assessment of the long-term bone stability after dental implantation is to be considered as a potential avenue for exploration. While the 1-month post-activation assessment showed nerve conduction parameters returning to preoperative levels, a longer follow-up period is required to fully evaluate the procedure’s neurosensory safety.

Vector-dictating distraction osteogenesis devices offer a predictable management option for vertically deficient ridges in the molar-premolar mandibular region, with attainment of the preferred crown-to-implant ratio and maintenance of the ideal bone orientation for favorable implant placement in relation to the occlusal loads. Furthermore, the integration of a meticulously fashioned virtual planning workflow allowed the execution of the procedure with predictable neurosensory safety and minimal nerve morbidity.

## Supplementary Information

Supplementary Material 1.

## Data Availability

All data generated or analysed during this study are included in this published article.

## Abbreviations

ADO: Alveolar Distraction Osteogenesis
CAD/CAM: Computer-Aided Designing/ Computer-Aided Manufacturing
C: ABI: Crown to Available Bone for Implantation Ratio
3D: Three-Dimensional
FDM: Fused Deposition Modelling
AP: Activation Period
MF: Mental Foramen
OL: Onset Latency
IAN: Inferior Alveolar Nerve
DICOM: Digital Imaging and Communications in Medicine
IAC: Inferior Alveolar Canal
STL: Standard Tessellation Language
VSP: Virtual Surgical Planning
LP: Latency Period
CP: Consolidation Period
ms: Milliseconds
CV: Conduction Velocity
