# Supplementary figures and images for: Roadmap of computer-guided bi-axial alveolar distraction osteogenesis in the posterior mandible: neurosensory safety of double scan virtual protocol
Source: BMC Oral Health. 2026 May 13;26:882. doi: 10.1186/s12903-026-08516-y (PMC13195843; doi:10.1186/s12903-026-08516-y)

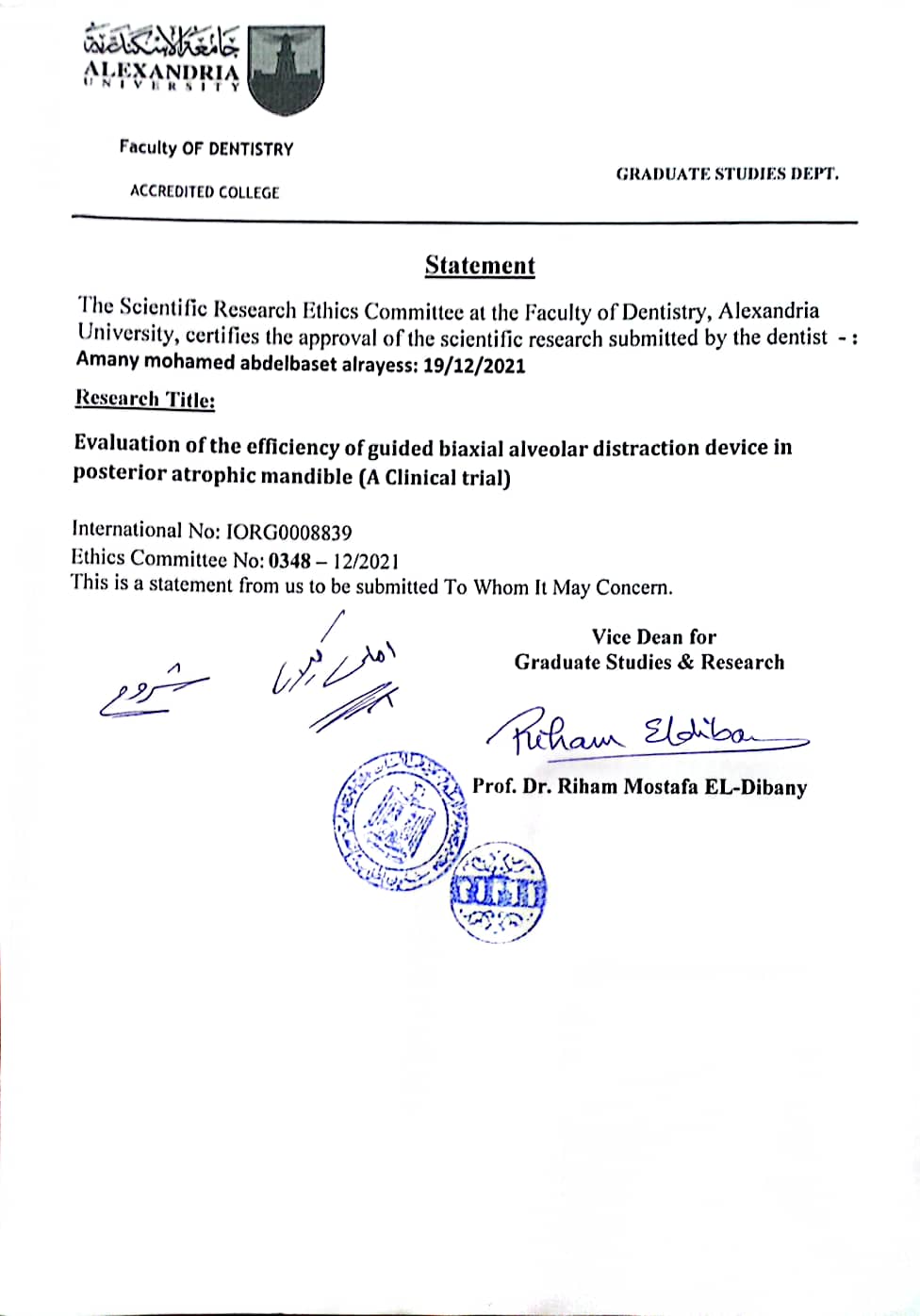

Supplement: Supplementary file 1 — Supplementary Material 1. [file 12903_2026_8516_MOESM1_ESM.docx]
